# The polymethoxy flavonoid sudachitin suppresses inflammatory bone destruction by directly inhibiting osteoclastogenesis due to reduced ROS production and MAPK activation in osteoclast precursors

**DOI:** 10.1371/journal.pone.0191192

**Published:** 2018-01-17

**Authors:** Yoko Ohyama, Junta Ito, Victor J. Kitano, Jun Shimada, Yoshiyuki Hakeda

**Affiliations:** 1 Division of Oral Anatomy, Meikai University School of Dentistry, Sakado, Saitama, Japan; 2 Division of Oral and Maxillofacial Surgery, Meikai University School of Dentistry, Sakado, Saitama, Japan; Charles P. Darby Children's Research Institute, UNITED STATES

## Abstract

Inflammatory bone diseases, including rheumatoid arthritis, periodontitis and peri-implantitis, are associated not only with the production of inflammatory cytokines but also with local oxidative status, which is defined by intracellular reactive oxygen species (ROS). Osteoclast differentiation has been reported to be related to increased intracellular ROS levels in osteoclast lineage cells. Sudachitin, which is a polymethoxyflavone derived from *Citrus sudachi*, possesses antioxidant properties and regulates various functions in mammalian cells. However, the effects of sudachitin on inflammatory bone destruction and osteoclastogenesis remain unknown. In calvaria inflamed by a local lipopolysaccharide (LPS) injection, inflammation-induced bone destruction and the accompanying elevated expression of osteoclastogenesis-related genes were reduced by the co-administration of sudachitin and LPS. Moreover, sudachitin inhibited osteoclast formation in cultures of isolated osteoblasts and osteoclast precursors. However, sudachitin rather increased the expression of receptor activator of NF-κB ligand (RANKL), which is an important molecule triggering osteoclast differentiation, and the mRNA ratio of RANKL/osteoprotegerin that is a decoy receptor for RANKL, in the isolated osteoblasts, suggesting the presence of additional target cells. When osteoclast formation was induced from osteoclast precursors derived from bone marrow cells in the presence of soluble RANKL and macrophage colony-stimulating factor, sudachitin inhibited osteoclastogenesis without influencing cell viability. Consistently, the expression of osteoclast differentiation-related molecules including c-fos, NFATc1, cathepsin K and osteoclast fusion proteins such as DC-STAMP and Atp6v0d2 was reduced by sudachitin. In addition, sudachitin decreased activation of MAPKs such as Erk and JNK and the ROS production evoked by RANKL in osteoclast lineage cells. Our findings suggest that sudachitin is a useful agent for the treatment of anti-inflammatory bone destruction.

## Introduction

Osteoclasts are the cells responsible for physiological and pathological bone resorption and belong to the monocyte/macrophage cell lineage [1]. Although many systemic hormones and local cytokines regulate osteoclast differentiation [2], the receptor activator of NF-κB (RANK) ligand (RANKL) and macrophage colony-stimulating factor (M-CSF) are the most important molecules in osteoclastogenesis. The signals of RANKL that is produced by bone marrow stromal cells or osteoblasts are introduced into osteoclast precursors, via a receptor of RANKL (RANK) on the plasma membrane of osteoclast lineage cells, leading to the activation of NF-κB and MAPKs [3–8]. The activation of the signaling molecules then induces the expression of NFATc1 [9, 10], a master transcription factor for osteoclast differentiation. Thereby, the expression of osteoclast differentiation-related molecules, including tartrate-resistant acid phosphatase (TRAP), cathepsin K and fusion-related proteins such as Atp6v0d2and DC-STAMP is induced [11–15].

Chronic autoimmune rheumatoid arthritis (RA), periodontitis and peri-implantitis are representative inflammatory bone diseases, accompanied by bone destruction by increasing the number of osteoclasts and functioning in collaboration with immune cells, osteoblasts and osteoclasts [16, 17]. Local inflammation in bone induces the production of pro-osteoclastogenic cytokines, including RANKL, tumor necrosis factor-α (TNF-α) and interleukins (ILs), such as IL-1β and IL-17 [16]. Simultaneously, local inflammation induces the production of reactive oxygen species (ROS) for host defense [18]. However, excessive production of ROS can damage molecules, such as DNA, proteins and lipids, in the cells surrounding the local inflammation site. On the other hand, ROS can also act as signaling transduction molecules involved in the regulation of many cellular events, such as angiogenesis, myogenesis and adipogenesis [19–21].

ROS have been suggested to stimulate osteoclast differentiation [22]. Increased ROS production has been linked to enhanced osteoclastogenesis in cell culture models [23, 24]. Therefore, limiting the excessive production of intracellular ROS has been assumed to prevent the extreme formation of osteoclasts induced by local inflammation. Extensive studies have examined the effects of various antioxidants, including biological compounds, such as glutathione, and compounds derived from natural foods, such as plant flavonoids, on osteoclast formation, inflammatory bone diseases and osteoporosis [25–28]. However, determining the direct effects of these antioxidants on bone resorption has been difficult, and the results have been inconclusive due to their diverse actions in a variety of cells. Under these circumstances, polymethoxy flavonoids derived from citrus fruits, such as nobiletin and tangeretin, have been demonstrated to exhibit anti-proliferative, apoptotic and anti-inflammatory effects on various cancer cells through their antioxidant actions [29–31]. Recently, sudachitin, which is a polymethoxy flavonoid derived from *Citrus sudachi*, has been reported to improve glucose and lipid metabolism by increasing mitochondrial biogenesis in skeletal muscle in addition to its potent antioxidant action [32–36]. However, the mechanism of action of sudachitin in bone metabolism and bone diseases remains unclear.

In this study, we found that sudachitin blocked LPS-induced inflammatory bone destruction by directly inhibiting osteoclast differentiation from osteoclast precursors without any effects on osteoblasts. Furthermore, sudachitin repressed the activation of Erk and JNK, which are pivotal signaling pathways for osteoclast differentiation, while simultaneously decreasing intracellular ROS production. Therefore, sudachitin may be a useful therapeutic agent for the treatment of inflammatory bone diseases.

## Materials and methods

### Mice

C57BL/6J mice were obtained from CLEA Japan, Inc. (Shizuoka, Japan). The experimental animal procedures were reviewed and approved by the Meikai University School of Dentistry’s animal care committee and conformed to relevant guidelines and laws. Animal sacrifice was humanely performed by cervical dislocation for adult mice and decapitation for neonatal mice.

### Antibodies and reagents

Sudachitin was obtained from Wako Pure Chemical Industries, Ltd. (Tokyo, Japan). LPS derived from *Escherichia coli* (O55:B5) was purchased from Sigma-Aldrich (St. Louis, MO). Recombinant human M-CSF was kindly provided by the Morinaga Milk Industry Co. (Tokyo, Japan). Recombinant murine soluble RANKL (sRANKL) and recombinant murine IL-1β were obtained from R&D Systems (Minneapolis, MN) and PeproTech (Rocky Hill, NJ), respectively. Prostaglandin E_2_ (PGE_2_) was obtained from Sigma-Aldrich. The anti-phospho-Erk1/2, anti-Erk1/2, anti-phospho-SAPK/JNK, anti-SAPK/JNK, anti-phospho-IκB, and anti-IκB antibodies were purchased from Cell Signaling Technology (Danvers, MA). The anti-c-fos and anti-NFATc1 antibodies were obtained from Santa Cruz Biotechnology (San Diego, CA). The anti-cathepsin K, anti-DC-STAMP (clone 1A2) and anti-Atp6v0d2 antibodies were purchased from BioVision (Mountain View, CA), MILLIPORE (Temecula, CA) and AVIVA Systems Biology (San Diego, CA), respectively.

### *In vivo* LPS-induced calvarial bone destruction model

The *in vivo* LPS-induced inflammatory calvarial bone destruction model was established as previously described [37]. Eight-week-old male C57BL/6J mice were injected with 100 μg LPS subperiosteally into the calvarial bone daily for 5 days. After 6 days, computed tomography (CT) scanning of the calvariae was performed using μCT (Skyscan 1172, Bruker, Billerica, MA) and reconstructed into a three-dimensional image. In addition, the calvarial bones were crushed in Buffer RLT Plus (Qiagen, Valencia, CA) using Polytron PT3100 (Kinematica AG), and the total RNA was subsequently extracted and used for cDNA synthesis using an RNeasy Mini plus kit (Qiagen).

### Osteoclast formation in a co-culture of osteoblasts from calvariae and osteoclast precursors from bone marrow cells

Osteoblasts were obtained from the calvariae of 3- to 7-day-old C57BL/6J mice by sequential digestion with 0.1% collagenase/0.2% dispase II in α-MEM, as previously described [37]. The cells released from the 3^rd^-5^th^ digestion were cultured for expansion in α-MEM/10% FBS and stored in liquid nitrogen as calvaria-derived osteoblasts. Osteoblasts (4000/well in a 96-multi-well plate) and osteoclast precursors (8000/well) derived from bone marrow cells pretreated with M-CSF (100 ng/ml) for 3 days were co-cultured in the presence of IL-1β (10 ng/ml) and PGE_2_ (10 μM) for 5–6 days. After culturing, the cells were fixed in 10% formalin and stained to detect the TRAP activity using a leukocyte acid phosphatase kit (Sigma-Aldrich). The osteoclast formation in the co-culture was evaluated by counting the TRAP-positive multi-nucleated cells (MNCs) per well. In addition, TRAP activity in the conditioned medium was measured using *p*-nitrophenyl phosphate as a substrate, as previously described [38].

### *In vitro* assay of osteoclastogenesis in bone marrow cells

The in vitro osteoclast formation was measured as previously described [39]. Briefly, bone marrow cells isolated from femora and tibiae are cultured for 3 days in α-MEM (ICN Biomedicals, Aurora, OH) containing 10% FBS and M-CSF (100 ng/ml) in a humidified atmosphere at 5% CO_2_. Following the removal of the non-adherent cells and the small population of stromal cells by washing the dishes with PBS and a subsequent incubation for 3 min in 0.25% trypsin/0.05% EDTA, the adherent monocytes were harvested by vigorous pipetting and used as osteoclast precursors. We used the high concentration (100 ng/ml) of M-CSF to obtain more osteoclast precursors efficiently, as previously reported [40]. The harvested osteoclast precursors were seeded in various tissue culture dishes and plates at an initial density of 2.5×10^4^/cm^2^ and cultured in α-MEM/10% FBS/M-CSF (20 ng/ml)/sRANKL (10 ng/ml) with or without various concentrations of sudachitin. The culture medium was exchanged with fresh medium every 2 days. TRAP-positive MNCs with more than 3 nuclei were considered osteoclastic cells and counted under a microscope. In addition, the enzymatic activity of TRAP in the conditioned medium was measured.

### Quantitative real-time PCR

The total RNA from the calvarial bones and cultured cells was reverse-transcribed using a High-capacity RNA-to-cDNA kit (Life Technologies) to produce cDNA. Quantitative real-time PCR was performed using the TaqMan Universal PCR Master Mix (Applied Biosystems, Foster City, CA) or Quantitative Tech SYBR Green PCR Master Mix (QIAGEN) on a GeneAmp 5700 Sequence Detection System (Applied Biosystems). The TaqMan primers for the indicated genes were obtained from Applied Biosystems and are listed in S1 Table. The relative quantification of the target mRNA expression was calculated and normalized to the amount of 18S rRNA. The primer sequences used for the quantitative RT-PCR performed with the SYBR Green PCR Master Mix are also listed in S1 Table.

### Western blotting analysis

After washing the cells with PBS, the cells were lysed in whole-cell lysis buffer [10 mM sodium phosphate (pH 7.5), 150 mM NaCl, 1% NP-40, 0.5% sodium deoxycholate, 0.1% SDS, 1 mM EDTA, 1 mM *p-*(aminoethyl)benzenesulfonyl fluoride (*p-*ABSF), 10 μg/ml leupeptin, 10 μg/ml pepstatin, and 10 μg/ml aprotinin] at 4°C, as previously described [37]. The whole-cell lysates were centrifuged at 12,000 × *g* for 10 min, and the supernatants were used for western blotting analysis. Samples of whole-cell lysates containing equal amounts of protein were subjected to SDS-PAGE, and the proteins that separated in the gel were subsequently electrotransferred onto PVDF membranes. After blocking with 5% skim milk, the membranes were incubated with the indicated antibodies, followed by a peroxidase-conjugated anti-mouse or anti-rabbit IgG antibody. The immunoreactive proteins were visualized, as previously described [37].

### Measurement of viable cells

The number of viable cells in the cultures of the osteoclast linage cells was estimated using a Cell Counting Kit-8 (CCK-8, Dojindo Molecular Technologies, Inc., Kumamoto), according to the manufacturer's instructions. The osteoclast precursors were cultured with M-CSF and sRANKL for 24 h and 48 h. Then, 10 μl of CCK-8 solution was added to the cultures. After a 1 h incubation, the absorbance of the culture was measured at 450 nm.

### Measurement of intracellular ROS

After culturing the osteoclast precursors in the presence of M-CSF and sRANKL with or without sudachitin for 24 h, we measured the concentration of intracellular ROS using an OxiSelect^TM^ Intracellular ROS Assay Kit (Green Fluorescence, Cell Biolabs, Inc., San Diego, CA), according to the manufacturer's instructions. The assay employs the cell-permeable fluorogenic probe 2’, 7’-Dichlorodihydrofluorescin diacetate (DCFH-DA). In brief, DCFH-DA is diffused into cells and is deacetylated by cellular esterases to non-fluorescent 2’, 7’-Dichlorodihydrofluorescin (DCFH), which is rapidly oxidized to highly fluorescent 2’, 7’- Dichlorodihydrofluorescein (DCF) by ROS [41]. After washing the cells twice with α-MEM, we added a DCFH-DA probe (100 μl)/α-MEM/10% FBS to the culture and incubated the cells for 30 min. Then, the probes were removed and washed with PBS. Subsequently, the cells were lysed in the cell lysis buffer. The concentration of DCF in the cell lysates was measured at an excitation of 480 nm and an emission of 530 nm using a standard solution of DCF.

### Statistical analysis

The data are presented as the mean ± standard error of the mean (SEM). The mean group values were compared by unpaired Student’s *t*-test, one-way ANOVA or two-way ANOVA; the significance of the observed differences was subsequently determined by post hoc testing using Tukey’s method or Bonferroni’s method. A *P*-value < 0.05 was considered significant.

## Results

### Sudachitin suppressed LPS-induced inflammatory bone destruction

**Fig 1:**
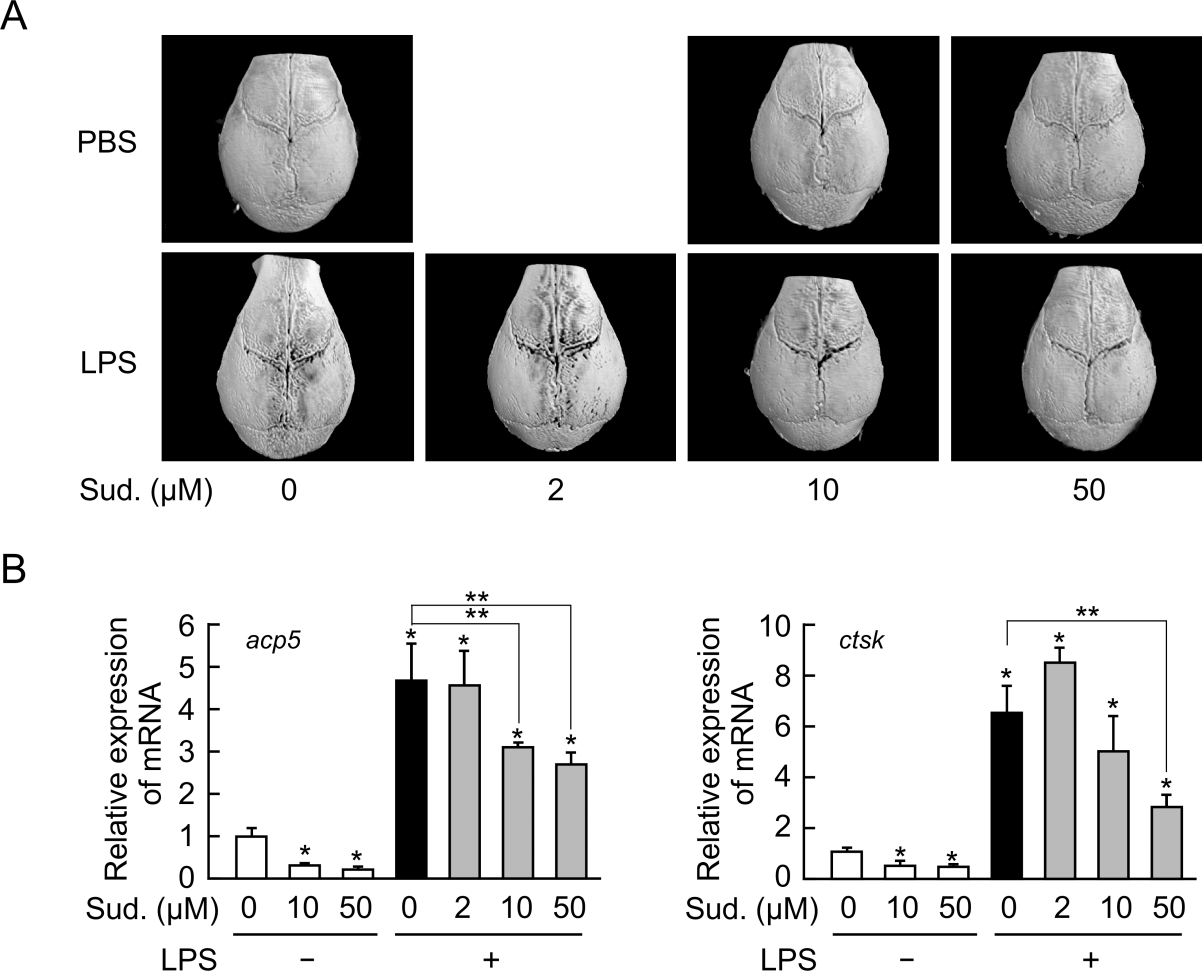
Sudachitin decreases inflammatory bone resorption and the expression of osteoclast differentiation-related genes in calvariae inflamed by an LPS injection. LPS (100 μg/day) or PBS with or without various concentrations of sudachitin (Sud.) was subperiosteally injected into the calvariae of mice once a day for 5 days *in vivo*. On day 6 after the initiation of the injections, the calvariae were removed, and the three-dimensional structures were determined by a μCT analysis (A). The total RNA was extracted from the injected region in calvariae, and the mRNA levels of osteoclast differentiation-related genes, such as *acp5* and *ctsk*, were measured by quantitative real-time RT-PCR (B). The presented values represent the mean ± SD (n = 3). *P < 0.05 *vs*. calvaria injected without LPS and sudachitin and **P < 0.05 *vs*. calvaria injected only with LPS.

The anti-inflammatory actions of sudachitin have been demonstrated by many *in vivo* and *in vitro* studies. Therefore, we first examined the effects of sudachitin on inflammatory bone loss using an LPS-induced *in vivo* model of inflammatory bone destruction. Following a subperiosteal LPS injection into the calvarial bones in mice, severe bone destruction was observed on both sides of the sagittal suture using three-dimensional μCT (Fig 1A). An injection of up to 50 μM sudachitin alone did not result in any of the irregular bone lesions observed following the LPS injection. However, an injection of sudachitin along with LPS markedly inhibited the bone loss induced by the LPS injection at a dose ranging from 10 and 50 μM (Fig 1A). Consistently, the LPS injection markedly increased the mRNA levels of osteoclast-differentiation related molecules, such as TRAP (*acp5*) and cathepsin K (*ctsk*), in the locally inflamed calvariae, and the simultaneous administration of sudachitin with LPS reduced these enhanced mRNA levels (Fig 1B). Thus, sudachitin strongly suppresses inflammatory bone destruction.

### Sudachitin inhibits osteoclastogenesis in a co-culture of osteoblasts and osteoclast precursors in the presence of pro-inflammatory factors

**Fig 2:**
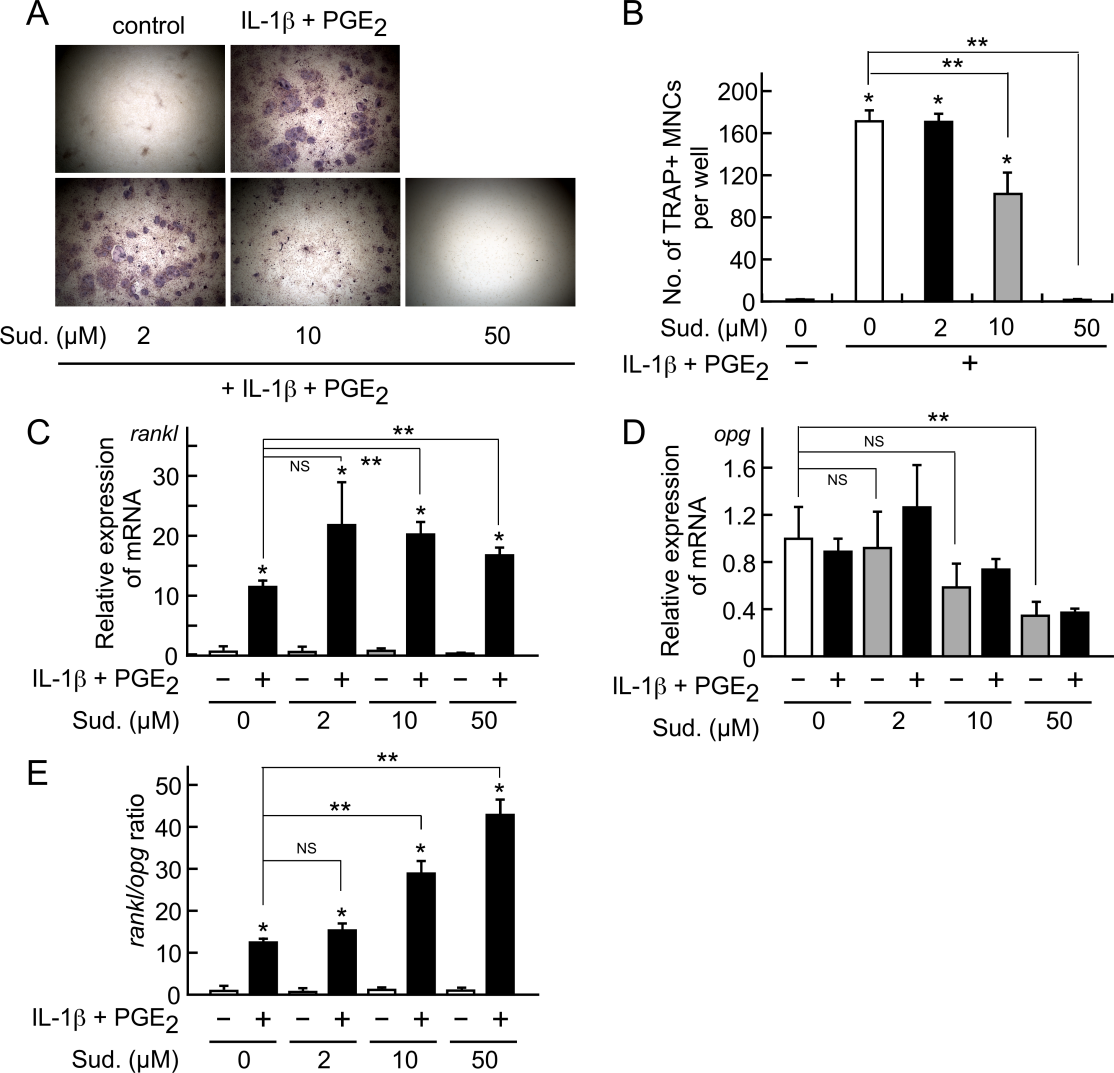
Effects of sudachitin on osteoclastogenesis in a co-culture of isolated osteoblasts and osteoclast precursors and the mRNA expression levels of RANKL and OPG in isolated osteoblasts. Isolated osteoblasts and osteoclast precursors were co-cultured in the presence of IL-1β (10 ng/ml) and PGE_2_ (10 μM) with various concentrations of sudachitin (Sud.) for 5 days. Then, the cells were stained for the detection of the TRAP activity. The photographs represent the TRAP-stained co-cultures (A). The TRAP-positive MNCs in the co-cultures were counted (B). The presented values represent the mean ± SD (n = 4). *P < 0.05 *vs*. co-culture in the absence of IL-1β and PGE_2_. **P < 0.05 *vs*. co-culture with IL-1β and PGE_2_ in the absence of sudachitin. In addition, the isolated osteoblasts were treated with various concentrations of sudachitin in the absence or presence of IL-1β (10 ng/ml) and PGE_2_ (10 μM) for 6 h. Then, the total RNA was extracted; the mRNA levels of RANKL (*rankl*, C) and OPG (*opg*, D) were measured by quantitative real-time RT-PCR, and the ratio of *rankl* mRNA/*opg* mRNA was calculated (E). The presented values represent the mean ± SD (n = 3). In C and E, *P < 0.05 *vs*. culture with IL-1β and PGE_2_ in the absence of sudachitin; **P < 0.05 *vs*. culture with IL-1β and PGE_2_ alone. In D, **P < 0.05 *vs*. culture without IL-1β, PGE_2_ and sudachitin. *NS* indicates that the difference is not significant.

Osteoclast formation proceeds following an interaction between osteoclast lineage cells and osteoblasts [37, 42]. Thus, we examined the effect of sudachitin on osteoclastogenesis in a co-culture of isolated osteoblasts and osteoclast precursors derived from bone marrow cells. In the co-culture, pro-inflammatory factors, such as IL-1β and PGE_2,_ induced osteoclast formation (Fig 2A and 2B). However, the simultaneous addition of 10 to 50 μM sudachitin with IL-1β and PGE_2_ inhibited osteoclast formation. The inhibitory effect of sudachitin was consistent with the effect observed following the LPS-induced inflammatory bone destruction in the calvariae.

To examine the mechanism underlying the inhibitory effect of sudachitin on osteoclast formation in the co-culture, we examined the mRNA expression levels of RANKL (*rankl*), which is a molecule that triggers osteoclast differentiation, and OPG (*opg*), which is an anti-osteoclast differentiation cytokine, in isolated osteoblasts (Fig 2C–2E). The *RANKL* mRNA expression level in the osteoblasts increased in response to the IL-1β and PGE_2_ treatment, and the enhanced level was maintained in the presence of sudachitin; however, sudachitin increased the mRNA level compared with that in the osteoblasts treated with IL-1β and PGE_2_ (Fig 2C). The *opg* mRNA expression level did not differ between the sudachitin-treated and untreated osteoblasts, while 50 μM sudachitin decreased the *opg* mRNA expression level (Fig 2D). In addition, sudachitin dose-dependently increased the mRNA ratio of *rankl* to *opg* (Fig 2E). Thus, the inhibitory effect of sudachitin on osteoclast formation in inflammatory bone destruction and the co-culture of osteoblasts and osteoclast precursors cannot be attributed to its action on osteoblasts.

### Sudachitin directly acts on osteoclast precursors and inhibits osteoclastogenesis

**Fig 3:**
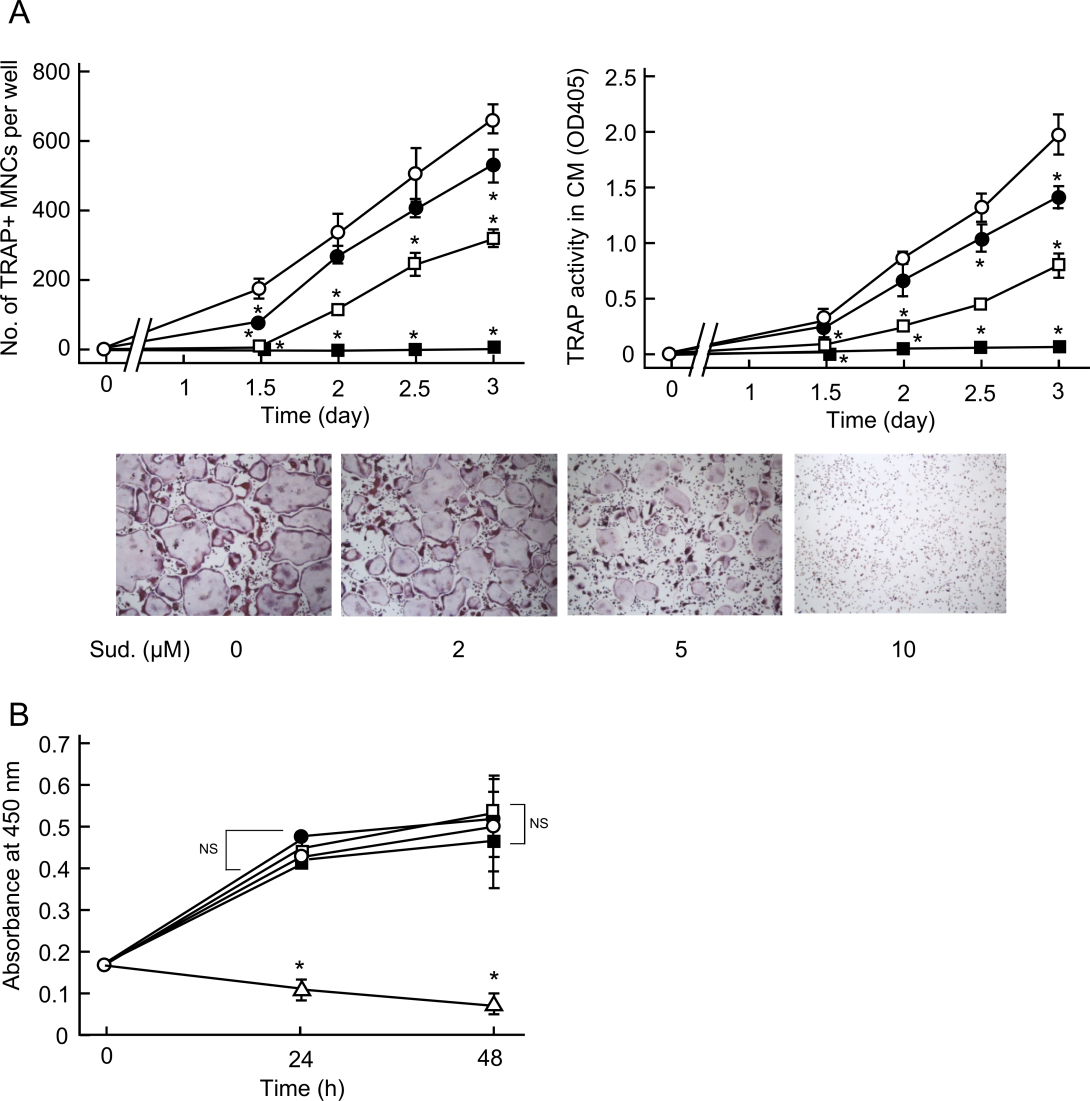
Sudachitin decreases osteoclastogenesis induced by RANKL in cultures of osteoclast precursors. Osteoclast precursors were treated with various concentrations of sudachitin (open circle, 0 μM; closed circle, 2 μM; open square, 5 μM; closed square, 10 μM) in the presence of sRANKL (10 ng/ml) and M-CSF (20 ng/ml) for the indicated times (A). At the end of the culture period, the cells were stained for the detection of TRAP activity, and the TRAP-positive MNCs were counted. In addition, the TRAP activity in the conditioned medium was measured. The presented values represent the mean ± SD (n = 4). *P < 0.05 *vs*. culture in the absence of sudachitin on each day. The photographs indicate the osteoclasts formed in the cultures on day 3. The magnification of the photographs was 40x. In another experiment, the osteoclast precursors were treated with sudachitin (open circle, 0 μM; closed circle, 2 μM; open square, 5 μM; closed square, 10 μM; open triangle, 30 μM)) in the presence of sRANKL and M-CSF for 24 h and 48 h. At each culture timepoint, the number of viable cells was measured. The presented values represent the mean ± SD (n = 4). *P < 0.05 *vs*. culture without sudachitin at each timepoint. *NS* indicates that the difference is not significant.

The above-mentioned results suggest that sudachitin plays other roles in addition to its role in osteoblasts. Thus, we next examined the effects of sudachitin on *in vitro* osteoclast formation from osteoclast precursors derived from bone marrow cells. In our culture system for *in vitro* osteoclast formation, the generation of TRAP-positive mononuclear preosteoclasts begins on day 1 after the addition of RANKL to the osteoclast precursor culture, and the preosteoclasts begin to fuse with each cell to generate TRAP-positive multinucleated osteoclasts between day 1.5 and day 3 (Fig 3A). Sudachitin at a concentration ranging from 2 and 10 μM time- and dose-dependently inhibited osteoclast formation and maturation (Fig 3A). In particular, at the 10 μM concentration, the TRAP-positive multinucleate cells barely formed in the culture. Consistently, sudachitin decreased the enzymatic activity of TRAP in the conditioned medium. Thus, sudachitin directly inhibits osteoclast differentiation. However, the number of viable cells in the presence of up to 10 μM sudachitin did not change, while a higher dose of sudachitin (30 μM) decreased cell viability (Fig 3B). Therefore, inhibition of osteoclastogenesis by sudachitin at low doses is not caused by a decrease in the cell viability. The effective sudachitin doses that inhibited *in vitro* osteoclast formation were lower than those that prevented inflammatory bone destruction *in vivo*. This discrepancy might have resulted from the local diffusion of sudachitin *in vivo*.

**Fig 4:**
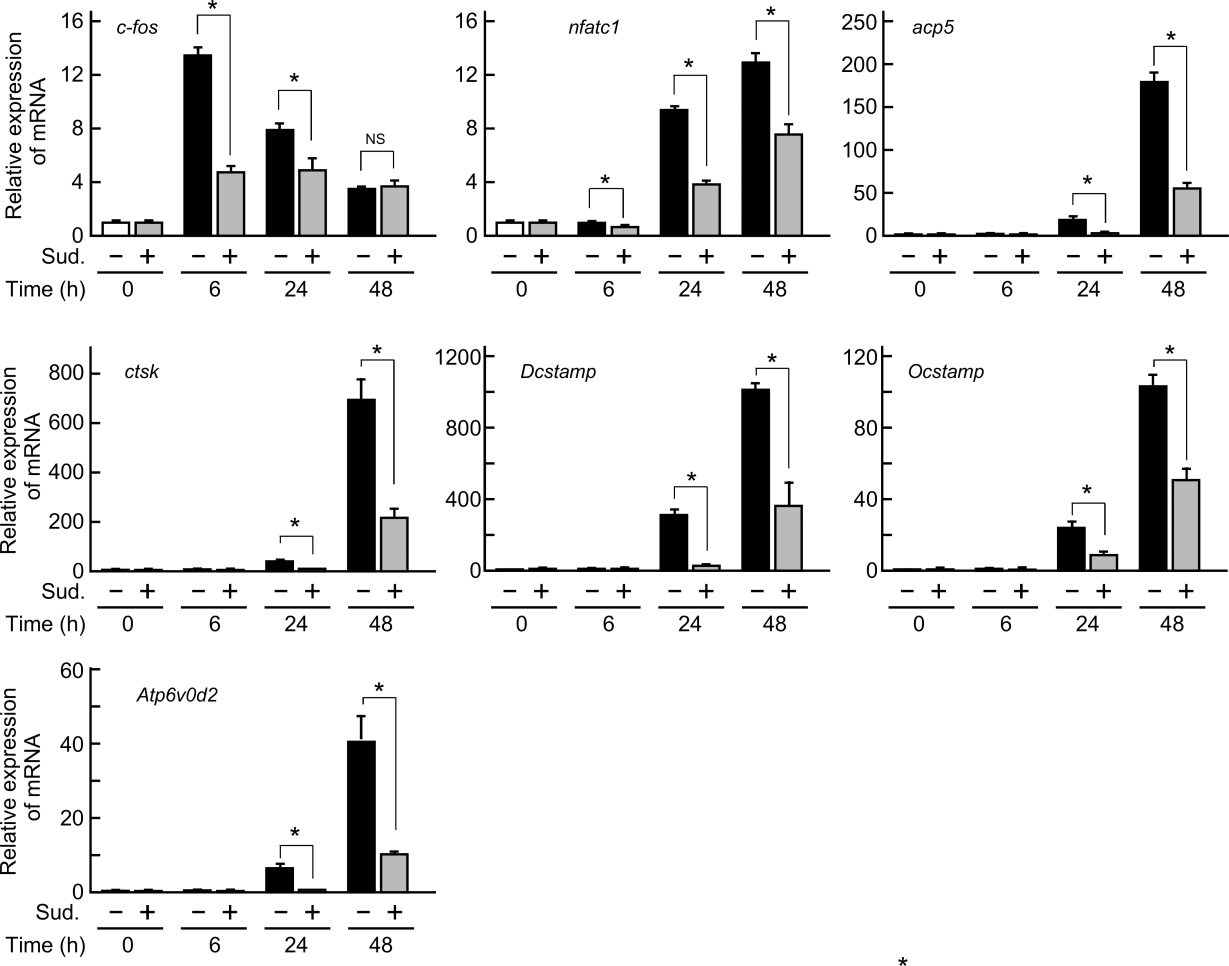
Inhibitory effect of sudachitin on the mRNA expression levels of osteoclast differentiation-related molecules in osteoclast lineage cells. Osteoclast precursors were cultured with M-CSF and sRANKL in the absence or presence of sudachitin (Sud., 10 μM) for the indicated durations. After culturing, total RNA was prepared and subjected to quantitative real-time PCR to determine the mRNA expression levels of *c-fos*, *nfatc1*, *acp5*, *ctsk*, *Dcstamp*, *Ocstamp* and *Atp6v0d2*. The values represent the mean ± SD (n = 3). *P < 0.05 *vs*. culture in the absence of sudachitin at each timepoint.

We next examined the expression of osteoclast differentiation-related molecules and the signaling pathways involved in osteoclastogenesis. The mRNA expression levels of *c-fos* and *NFATc1*, which are crucial transcription factors for osteoclast differentiation, *TRAP* (*acp5*) and *cathepsin K* (*ctsk*), which are functional enzymes for bone resorption, and *DC-STAMP*, *OC-STAMP* and *Atp6v0d2*, which are integral proteins for the cell-cell fusion of preosteoclasts, markedly increased in response to the sRANKL/M-CSF treatment during osteoclast formation (Fig 4). The enhanced mRNA levels decreased in the presence of sudachitin (10 μM) at all timepoints of the culture (Fig 4).

**Fig 5:**
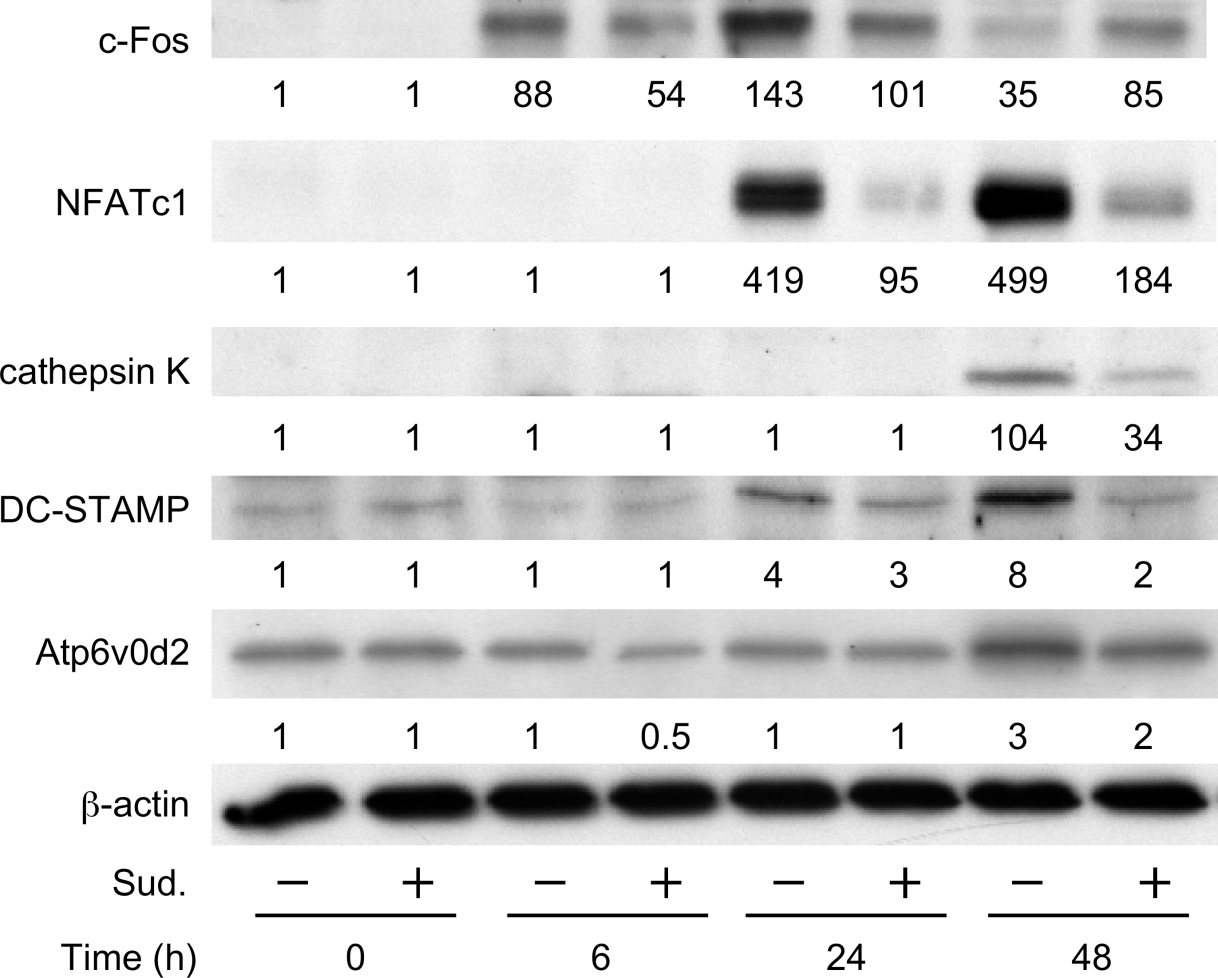
Inhibitory effect of sudachitin on the protein levels of osteoclast differentiation-related molecules in osteoclast lineage cells. Osteoclast precursors were cultured with M-CSF and sRANKL in the absence or presence of sudachitin (Sud.,10 μM) for the indicated durations. After culturing, whole-cell lysates were prepared and subjected to a western blotting analysis to determine the protein levels of c-Fos, Nfatc1, cathepsin K, DC-STAMP and Atp6v0d2. β-Actin was used as an internal control. The values presented below the images indicate the relative level of each protein compared with the level of β-actin.

Consistent with the inhibitory effects of sudachitin on the mRNA expression levels in the osteoclast lineage cells, sudachitin decreased the increased protein levels of these osteoclast differentiation-related molecules including c-Fos, NFATc1, cathepsin K, DC-STAMP and Atp6v0d2 during osteoclast formation stimulated by sRANKL and M-CSF (Fig 5).

### Sudachitin suppresses the production of intracellular ROS and activation of MAPKs in osteoclast lineage cells

**Fig 6:**
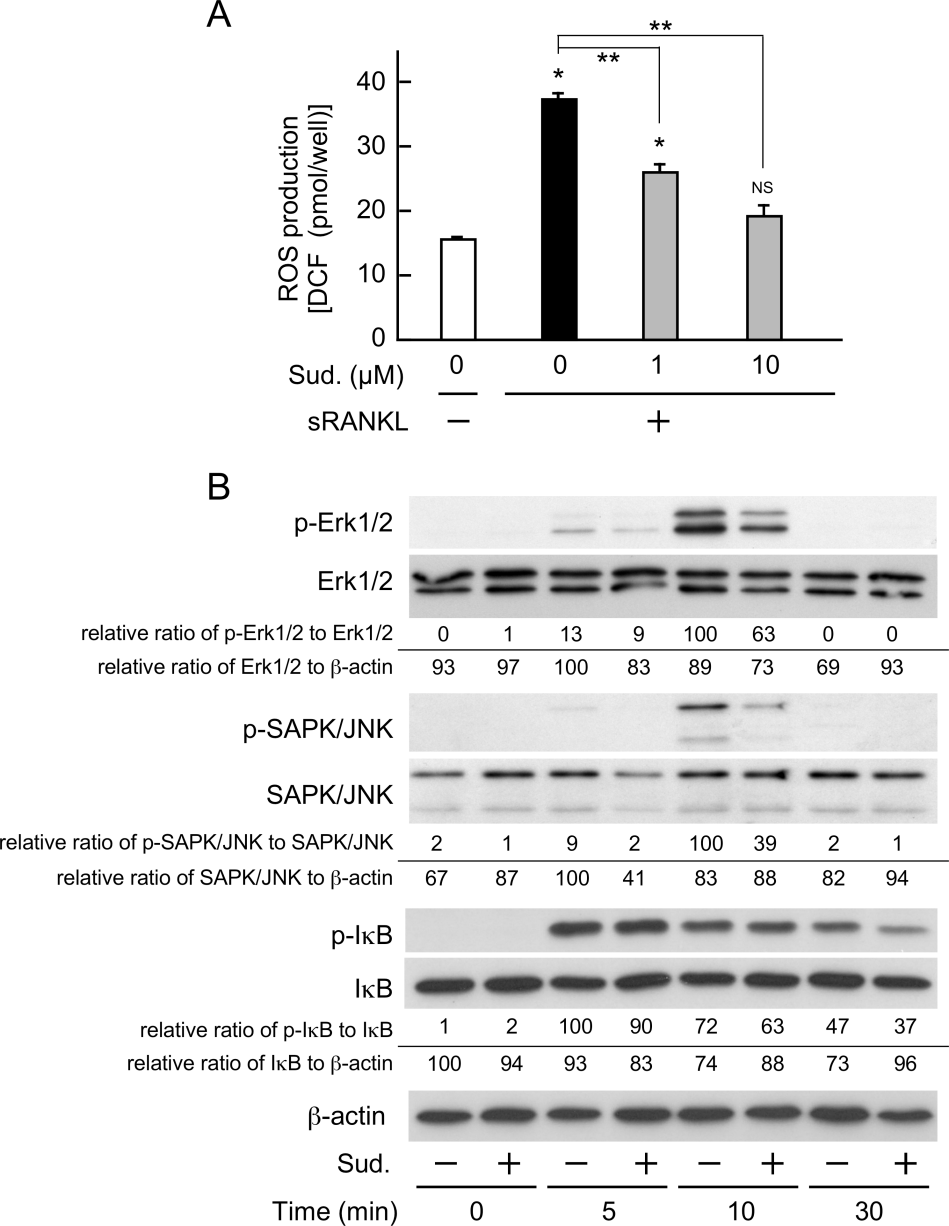
Sudachitin suppresses the production of intracellular ROS and activation of MAPKs in osteoclast lineage cells. Osteoclast precursors were treated with or without sudachitin (Sud.) in the presence of M-CSF and/or sRANKL for 24 h. Then, the concentration of ROS in the cells was measured (A). The presented values represent the mean ± SD (n = 5). *P < 0.05 and *NS* (not significant) *vs*. culture with M-CSF alone; **P < 0.05 *vs*. culture with sRANKL and M-CSF. In addition, osteoclast precursors were cultured in the absence or presence of sudachitin (10 μM) in α-MEM/10% FBS for 3 h. Then, the cells were treated with sRANKL (20 ng/ml) with or without sudachitin in α-MEM/10% FBS for the indicated times. Subsequently, whole-cell lysates were prepared and subjected to a western blotting analysis to measure phosphorylated and unphosphorylated Erk1/2, SAPK/JNK, and IκB-α levels. The values presented below the images indicate the relative ratios of the total band intensity of phosphorylated Erk1/2 (p44 and p42) and SAPK/JNK (p46 and p54) to the total band intensity of non-phosphorylated Erk and SAPK/JNK, respectively. In case of IκB-α, the values are the relative ratio of phosphorylated IκB-α non-phosphorylated IκB-α. In addition, total amounts of Erk1/2 (p44 and p42), SAPK/JNK (p46 and p54) and IκB-α were also compared with the level of β-actin.

Osteoclast differentiation has been associated with an elevated production of intracellular ROS [23, 43, 44]. Furthermore, sudachitin has been shown to exert antioxidant effects in the body [34]. Therefore, to explore whether the inhibitory effect of sudachitin on osteoclastogenesis is related to the intracellular production of ROS, we determined the concentration of intracellular ROS in osteoclast lineage cells treated with sRANKL and/or sudachitin. As shown in Fig 6A, when osteoclast precursors were treated with sRANKL for 24 h, the intracellular ROS content was 2.5-fold higher than that in the cells treated with M-CSF alone. However, the simultaneous addition of sudachitin with sRANKL dose-dependently attenuated the elevated intracellular ROS content. The ROS content in the cells treated with sRANKL plus 10 μM sudachitin was equivalent to that in the cells treated with M-CSF alone. Thus, sudachitin inhibited the intracellular production of ROS during osteoclast formation.

Finally, we examined the effects of sudachitin on the activation of osteoclast differentiation-signaling pathways, such as NF-κB and MAPKs, including Erk and JNK. The activation of these pathways was evoked by the addition of sRANKL in the short term. However, pretreatment with sudachitin reduced the activation of Erk and JNK (Fig 6B). The activation of NF-κB stimulated by sRANKL is also slightly decreased by the sudachitin-pretreatment, whereas the reduction was less than those of Erk and JNK (Fig 6B).

## Discussion

In this study, we demonstrated that sudachitin, which is a polymethoxy flavonoid, suppresses inflammatory bone destruction induced by an LPS injection in the calvariae. The inhibition by sudachitin could be attributed to the direct inhibition of osteoclastogenesis from osteoclast precursors because sudachitin reduced osteoclast formation from osteoclast precursors in response to RANKL *in vitro* and sudachitin increased the ratio of *RANKL* to *Opg* in the osteoblasts. Furthermore, sudachitin suppressed the intracellular ROS production and the activation of MAPKs, including Erk and JNK, both of which are involved in osteoclast differentiation. These inhibitory activities could be associated with a decrease in osteoclast formation. Thus, sudachitin could be a useful agent for the treatment of inflammatory bone destruction.

ROS are mainly classified into the following four types: hydroxyl radicals, nitric oxide, superoxide anions and hydrogen peroxide. Although the role of ROS in osteoclast formation has been reported since 1990, the ROS type that is important for osteoclastogenesis has not been determined. The ROS production in osteoclast lineage cells has been demonstrated to be mediated by NADHP oxidases, which consist of five isoforms, i.e., Nox1 to Nox5. Furthermore, many NOX organizers, NOX activators and small GTPases participate in regulating the enzymatic activities of the NADPH oxidases [45]. Although NOX1^-/-^, NOX2^-/-^ and NOX3 mutant mice do not demonstrate bone abnormalities [44, 46, 47], global Nox4-knockout mice display a higher trabecular bone density and reduced numbers and markers of osteoclasts *in vivo* [44]. In particular, *ex vivo* experiments using NOX4^-/-^ osteoclast precursors showed a reduction in osteoclastogenesis that is consistent with the down-regulation of intracellular ROS production. In addition, the NOX inhibitors GKT137928 and GKT137831 both rescued the bone loss induced by ovariectomy [44]. These observations highlight the pivotal role of intracellular ROS catalyzed by NOX4 in osteoclast differentiation and function. The present study also indicates the importance of intracellular ROS in osteoclastogenesis because sudachitin, which has antioxidant properties, strongly inhibited inflammatory bone destruction and osteoclast formation, changes accompanied by a reduction in the intracellular ROS content.

Although many studies have demonstrated the effects of antioxidant compounds on osteoclast differentiation associated with the intracellular redox status, the effects have not always been consistent and remain controversial. Glutathione (GSH) is a representative antioxidant, and cellular redox status is defined by the balance between oxidants and antioxidants, in particular the GSH/oxidized GST (GSSG) ratio. Romagnoli et al demonstrated that a low GSH/GSSG ratio downregulated OPG expression in human osteoclasts, resulting in an increase in the ratio of RANKL to OPG and indicating that GSH/GSSG redox coupling could affect osteoclastogenesis [48]. However, these authors did not directly determine the influence of the GSG/GSSG change on osteoclast formation. Le Nihouannen et al found that the addition of ascorbic acid to a culture of osteoclast precursors in the presence of RANKL decreased osteoclast formation, which was consistent with the decrease in the ratio of GSH/GSSG [49]. However, several studies have reported an inhibitory effect of ascorbic acid on osteoclastogenesis [50], indicating the difficulty of controlling the intracellular redox status.

The efficacy of natural food-derived antioxidants in osteoclastic bone resorption has also been extensively studied [51]. Recent epidemiological studies have shown that flavonoid consumption has a stronger association with bone integrity than general fruit and vegetable consumption. Epigallocatechin gallate and its polymerized theaflavin digallate, which are contained in green and black tea, exert suppressive effects on all processes of osteoclast differentiation, including osteoclast precursor generation from bone marrow cells, osteoclast differentiation from osteoclast precursors and the maturation of multinucleated functional osteoclasts [52–54]. More recently, polymethoxy flavonoids contained in citrus fruits, such as nobiletin and tangeretin, have attracted attention due to their diverse physiological activities, such as the repression of carcinogenesis and cancer growth, anti-atherosclerosis activity, and the improvement of dyslipidemia and anti-inflammatory diseases. Although the sudachitin used in this study is also a polymethoxy flavonoid and has previously shown stronger physiological activities than nobiletin and tangeretin, its effects on bone metabolism were revealed for the first time in this study. Our findings show that the action of sudachitin in the suppression of osteoclast formation is more prominent than that of other polymethoxy flavonoids. In addition, this suppression was attributed to the direct inhibition of osteoclast differentiation from osteoclast precursors without acting on osteoblasts. Therefore, although the inhibition of osteoclast formation by nobiletin and tangeretin has been previously reported to be associated with the suppression of intracellular PGE_2_ production in osteoblasts [31], our study indicates that the primary cells targeted by sudachitin are osteoclast lineage cells.

The elevation of intracellular ROS content causes the activation of MAPKs, such as Erk, p38 MAPK and JNK, in various cells [55–57]. Consistently, in this study, RANKL, which is a molecule that triggers osteoclast differentiation, induced the activation of Erk and JNK and increased ROS; simultaneously, sudachitin also inhibited the elevation of ROS and the activation of MAPKs. In contrast, sudachitin hardly affects the activation of NF-κB evoked by RANKL in osteoclast precursors, suggesting that the primary action target of sudachitin is MAPK rather than NF-κB.

Various types of antibodies targeting pro-inflammatory cytokines have currently been used for the treatments of inflammatory bone destruction such as RA, resulting in significant improvements in clinical scores [58–60]. However, in some cases of treatment with an anti-RANKL antibody, hypocalcemia developed in patients with severe renal dysfunction [61]. Moreover, treatments using these antibodies are extremely expensive. As previously mentioned, sudachitin has a variety of useful biological activities and is relatively inexpensive. In addition, as bone loss induced by not only inflammation but also ovariectomy [44] is associated with the elevation of intracellular ROS production, our findings suggest that use of sudachitin may lead to a possible therapeutic approach for various bone diseases including postmenopausal osteoporosis and inflammatory bone destruction.

## Conclusions

Sudachitin, which is a polymethoxy flavonoid, blocked LPS-induced inflammatory bone destruction by directly inhibiting osteoclast differentiation from osteoclast precursors. Furthermore, sudachitin repressed the activation of Erk and JNK, which are pivotal signaling pathways for osteoclast differentiation associated with a decrease in intracellular ROS production. Therefore, sudachitin is a useful therapeutic agent for inflammatory bone resorption.

## Supporting information

S1 Table Primers and probes used in quantitative RT-PCR. (DOCX)
