# Supplementary material for: The polymethoxy flavonoid sudachitin suppresses inflammatory bone destruction by directly inhibiting osteoclastogenesis due to reduced ROS production and MAPK activation in osteoclast precursors
Source: PLoS One. 2018 Jan 17;13(1):e0191192. doi: 10.1371/journal.pone.0191192 (PMC5771597; doi:10.1371/journal.pone.0191192)
Supplement: S1 Table — (DOCX) [file pone.0191192.s001.docx]

**S1 Table. Primers and probes used in quantitative RT-PCR.**

Taqman probe ID (Applied Biosystems)

*Acp5* Mm00475698_m1

*Ctsk* Mm00484039_m1

*c-fos* Mm00487425_m1

*Nfatc1* Mm00479445_m1

*Rankl* Mm00441906_m1

*Opg* Mm01205928_m1 *Dcstamp* Mm04209236_m1

*Ocstamp* Mm00512445_m1

*Atp6v0d2* Mm01222963_m1
18s rRNA　　　　 4319413E

Primer sequences for quantitative RT-PCR using SYBR Green PCR Master Mix

*Rankl* forward 5’-CAAGCTCCGAGCTGGTGAAG-3’

*Rankl* reverse 5’-CCTGAACTTTGAAAGCCCCA-3’

*Opg* forward 5’-AAGAGCAAACCTTCCAGCTGC-3’

*Opg* reverse 5’-CACGCTGCTTTCACAGAGGTC-3’
